# Job Strain and Self-Reported Insomnia Symptoms among Nurses: What about the Influence of Emotional Demands and Social Support?

**DOI:** 10.1155/2015/820610

**Published:** 2015-10-18

**Authors:** Luciana Fernandes Portela, Caroline Kröning Luna, Lúcia Rotenberg, Aline Silva-Costa, Susanna Toivanen, Tania Araújo, Rosane Härter Griep

**Affiliations:** ^1^National School of Public Health (ENSP/Fiocruz), Avenida Brasil 4365, 21040-360 Rio de Janeiro, RJ, Brazil; ^2^Health and Environmental Education Laboratory, Oswaldo Cruz Institute (IOC/Fiocruz), Avenida Brasil 4365, 21040360 Rio de Janeiro, RJ, Brazil; ^3^Centre for Health Equity Studies (CHESS), Stockholm University and Karolinska Institute, Sveaplan, Sveavägen 160, Floor 5, 106-91 Stockholm, Sweden; ^4^Department of Health, State University of Feira de Santana, R. Cláudio Manoel da Costa 74/1401, Canela, 40110-180 Salvador, BA, Brazil

## Abstract

Job strain, derived from high psychological demands and low job control, is associated with insomnia, but information on the role of emotional demands and social support in this relationship is scarce. The aims of this study were (i) to test the association between job strain and self-reported insomnia symptoms, (ii) to evaluate the combination of emotional demands and job control regarding insomnia symptoms, and (iii) to analyze the influence of social support in these relationships. This cross-sectional study refers to a sample of nurses (*N* = 3,013 and *N* = 3,035 for Job Strain and Emotional demand-control model, resp.) working at public hospitals in Rio de Janeiro, Brazil. Data were collected through a self-report questionnaire. The prevalence of insomnia symptoms was 34.3%. Job strain was associated with increased odds for insomnia symptoms (OR: 2.20); the same result was observed with the combination of emotional demands and low job control (OR: 1.99). In both models, the inclusion of low social support combined with high demands and low job control led to increased odds for insomnia symptoms, compared to groups with high social support from coworkers and supervisors. Besides job strain, the study of emotional demands and social support are promising with regards to insomnia symptoms, particularly among nurses.

## 1. Introduction

Insomnia is known as a relevant public health problem, with a complex etiology [1, 2]. Sleep complaints can have negative effects on the immune system and metabolism [3] as well as various health issues such as depression [4], hypertension [5], and coronary heart diseases [6]. Employees' sleep disturbances can have significant effects on organizations' performances due to impairments in concentration, communication skills, decision-making, and flexible thinking. Sleep disturbances may also lead to reduced job motivation and poor leadership qualities [7].

Previous researches identified several risk factors for insomnia. On one hand, there were individual factors such as female gender, increasing age, low body mass index (BMI), low socioeconomic status, marital status, and presence of physical or mental illness [8, 9]. On the other hand, personal behaviours, such as frequent use of alcohol [10] and dietary patterns [11], were also documented in the literature. In addition, work-related factors including psychosocial effects of the work environment (such as job satisfaction, overcommitment, effort-reward-imbalance, hectic work, physically strenuous work, shift work, job insecurity, organizational injustice, low employment opportunities, and job strain) can also be counted as risk factors for insomnia [7, 12–17].

The job strain model by Karasek assesses psychosocial job stress by taking psychological demands and the job control into account. Psychological demands arise particularly from time pressure, amount of work, and conflicting work instructions and job control or decision latitude describes the level of workers' control over the performance of their jobs. Job strain results from the interaction of high psychological demands and low job control [18, 19].

In a further approach to psychosocial stress at work by Karasek, emotional demands [20] and social support from supervisors and coworkers [18] were evaluated in the job strain model. Emotional demands have an impact on feelings or emotions and are strongly related to interpersonal relationships, including caring and concern for others [20]. Social support by supervisors and colleagues helps workers to appreciate their own value and competencies and enables them to cope with upcoming demands and difficult situations [19]. Social support, as defined by Karasek and Theorell, is “an overall level of helpful social interaction available on the job from both coworkers and supervisors” [19].

More recently, the importance of including emotional demands in the job strain model has been emphasized by several authors, particularly considering jobs involving interaction with clients [21–23]. In the context of these discussions, van Vegchel et al. [24] argue that the demand-control-support model would give an oversimplified image for human service work, proposing that emotional demands are an essential complement to psychological demands, as workload. Actually, emotional demands have been associated with physical symptoms, as low back pain [25], psychological outcomes [26], and occupational injuries [23].

A number of investigations have reported increase of sleep disturbances or insomnia due to high demands, low job control, and job strain [12, 13, 16, 27]. In addition, some authors have investigated the role of social support for insomnia [14, 15, 28, 29] or the buffering effect of job control and social support against insomnia [7, 15, 17, 30, 31]. Some studies have associated social support but not job strain with insomnia. In contrast, some studies did not show a buffering effect of social support and high job control on better sleep quality [5, 30].

In the present study the relationship between job strain and self-reported insomnia symptoms among nurses is dealt with through two aspects that deserve further investigation: Firstly, the partly conflicting study results regarding the role of social support by itself and combined with the job strain model. Secondly, a detailed study of emotional demands since this construct is relatively recent considering the job strain model. To our knowledge, there have been no studies focusing on the combination of emotional demands and job control in relation to insomnia, although the relations between sleep and other approaches of emotional demands have already been addressed [32–34]. Job stress among nurses is approached in the present study by using two models: (i) the job strain model, derived from psychological demands and job control and (ii) the combination of job control with emotional demands. This study integrated social support into the models under the assumption that the prevalence of self-reported insomnia symptoms is higher among nurses with high psychological and emotional job demands and with low decision latitude and low social support, compared with nurses without these stressors. Three objectives guided the study: (i) to investigate the association between the job strain model and self-reported insomnia symptoms, (ii) to evaluate the combination of emotional demands and job control regarding the association with self-reported insomnia symptoms, and (iii) to analyze the influence of social support on these associations.

## 2. Methods

### 2.1. Subjects

Nurses from the 18 largest public hospitals (with over 150 beds) in the city of Rio de Janeiro, Brazil, participated in this cross-sectional study. Each nurse was contacted personally by a team of interviewers (in most cases, nurses themselves), who explained the objectives of the study and invited them to participate. After signing the consent form, the nurses received a comprehensive self-report questionnaire to complete. The questionnaire was divided into three blocks, corresponding to (i) variables related to work (number of jobs, weekly work hours, work schedule, intention to leave the profession, recovery after work scale, effort-reward imbalance scale, psychological and emotional demands, job control, and social support from workers and from colleagues), (ii) behaviours related to health and lifestyle (self-reported diagnosis of hypertension and cardiovascular diseases, sickness absenteeism, sleep duration, self-reported insomnia symptoms, sleep satisfaction, weight, height, practice of physical activity, smoking, consumption pattern of alcoholic beverages, coffee consumption, common mental disorder), and (iii) sociodemographic data and information on the domestic sphere (school degree, income, marital status, number and age of children, type of domestic duties, and number of hours of domestic work). Data collection was carried out from March 2010 to November 2011. Trained reviewers entered, reviewed, and coded the information contained in the returned questionnaire into a database using the software Microsoft Access 2010.

### 2.2. Study Variables

#### 2.2.1. Self-Reported Insomnia Symptoms

Individuals that reported insomnia symptoms were defined as those who answered* often *or* always* to any of the questions concerning “Difficulty to sleep or get to sleep,” “Waking up during the night (more than three times),” or “Waking up too early in the morning and having trouble getting back to sleep” [7]. The response categories for the questions were “Never,” “Rarely (a few times per year),” “Sometimes (several times per month),” “Often (several days per week),” and “Always (every day).”

#### 2.2.2. Job Stress

Perceived stress at work was measured by the Portuguese version of the Job Content Questionnaire 2.0 (JCQ 2.0) [35]. This scale measures psychological demands, job control, and social support from supervisors and coworkers. It also includes questions on emotional demands, which were evaluated by a three-item scale regarding being confronted with emotionally demanding work, having to hide one's emotions during work or demanding a lot of negotiation, discussion, or understanding with others.

The response categories were a Likert-scale ranging from 1 (strongly disagree) to 4 (strongly agree) and were reverse-coded afterwards if necessary. The following were the Cronbach's alpha coefficients: psychological demands (0.71), emotional demands (0.59), job control (0.75), supervisor support (0.74), and coworker support (0.81).

Two stress models were analyzed: the job strain, based on psychological demands and job control, and the emotional demand-control model. The first step in the analysis was the description of each individual dimension, which was based on the categorization into approximately equal-sized terciles. Second, to compute the job strain both psychological demands and job control were dichotomized at the median value into high and low groups, leading to four job categories: low-strain, passive job, active job, and high-strain job [19]. A similar procedure was conducted for the emotional demand-control model. In a further step, the social support categories (low and high) were added to the low-strain and the high-strain quadrants of the job strain model, leading to four combinations: low-strain with high social support (reference group), low-strain with low social support, high-strain with high social support, and high-strain with low social support. An analogous procedure was performed for the emotional demand-control model, also resulting in four combinations. In this case, the reference group comprised workers with low emotional demand-high job control plus high social support.

#### 2.2.3. Covariates

Relevant covariates associated with both self-reported insomnia symptoms and job strain were considered in the present study as possible confounders [2, 15–17]. Sociodemographic covariates included age, sex,* per capita* income, marital status (married/cohabiting, divorced/separated, or never married/cohabiting), and children less than six years old. Work-related variables included night work (current night-worker, former night-worker, or never worked nights), as well as working hours per week (<40, 40–59, and ≥60). Health-related factors were the risk of alcohol consumption (frequency and dose of alcohol per week based on the recommendation of the National Institute on Alcohol Abuse and Alcoholism) [36], smoking habits (smoker, ex-smoker, and nonsmoker), and coffee intake (numbers of cups of coffee per day). Body mass index (BMI) was defined by self-reported weight (kg)/height (m^2^). BMI was categorized as underweight (<18.49), normal weight (18.50–24.99), overweight (25.00–29.99), and obese (≥30.00).

### 2.3. Statistical Analysis

Descriptive analyses of sociodemographic variables as well as variables related to work and health were based on chi-square tests. Multivariate logistic regression analyses, with 95% confidence intervals (95% CI), were used to examine the associations between each model of job stress and self-reported insomnia symptoms. Both crude and adjusted odds ratios were presented (significance at *p* < 0.05). All data were analyzed using the software IBM Statistical Package for the Social Sciences version 19.0 (IBM SPSS).

## 3. Results

The number of completed questionnaires returned was 3,229 (82.7% of the total number of nurses) and these were part of the analysis for this present paper. The 675 (17.3%) who did not participate were due to refusals (*n* = 478), nurses being unavailable across multiple visits over two months (*n* = 128), and absences because of holidays (*n* = 69). Considering that the analyses are based on scales, data from participants who did not answer a single question were excluded from databank, resulting in a final sample size of 3013 workers for the analysis of job strain, and 3035 for the emotional demand-control model. We performed a sensitivity analysis in which we assumed that persons with missing information for job strain did not have an exposure in that category (i.e., they were classified in the low strain group). This procedure was adopted to test the influence of missing data on the results [37]. Results of the multivariate logistic regression analysis showed that the direction and the strength of the associations between job strain and self-reported insomnia symptoms were nearly identical as the previous ones. The same result was observed as regards the emotional demand-control model (data not shown). Thus, the loss of these participants did not alter significantly any of the above-reported results.

The participants were predominantly female (87.3%) with mean age of 39.9 years (±10.0; range: 22 to 68 years). Overall the prevalence of self-reported insomnia symptoms was 34.3%. The highest prevalence of self-reported insomnia symptoms was observed for the oldest groups, women, married or divorced, and participants with children under six years old. Persons working 40 hours per week or less and former night workers showed higher self-reported insomnia symptoms prevalence compared with their counterparts. Participants with a very low or high BMI and who were not physically active had a higher prevalence of self-reported insomnia symptoms. In addition, the prevalence of self-reported insomnia symptoms was highest among high-risk drinkers, current smokers, and persons with a coffee intake of more than three cups per day. Neither race nor education level was associated with self-reported insomnia symptoms (Table 1).

Table 2 shows that odds ratio corresponding to all stress dimensions presented a gradient. Thus, compared with low demands, adjusted OR for medium and high psychological demands were 1.33 and 1.82, respectively. The corresponding values for emotional demands were 1.19 and 1.59, respectively. A gradient from higher to lower degree of job control and social support were also observed. Low job control corresponded to OR = 1.40 (CI 95% 1.14–1.72) and low social support corresponded to OR = 1.59 (CI 95% 1.29–1.95).

According to Table 3, participants with high demands and low control showed a higher prevalence of self-reported insomnia symptoms than the respective reference groups both regarding job strain (OR = 2.20) and the emotional demand-control model (OR = 1.99). For both models, the inclusion of low social support resulted in an increased prevalence of self-reported insomnia symptoms, compared with the combination with high social support, particularly for the emotional demand-control model, with an increase from OR = 1.41 to OR = 2.47.

## 4. Discussion

An original contribution of this study is the analysis of the combination of high emotional demands and low job control, which was shown to be associated with self-reported insomnia symptoms. Emotional demands have recently been added to the 2.0 version of JCQ [35], but studies on this construct linked to the demand-control model are yet scarce, and no one was identified dealing with self-reported insomnia symptoms. The results seem to be plausible given that emotionally demanding occupations are related to health outcomes and burnout [26], although the underlying mechanisms are still unknown [22]. Actually, nursing is a profession with high emotional demands throughout the daily working routine [21, 38]. Results on the emotional demand-control model follow the ones obtained in previous research on the relationship between job strain and self-reported evaluations of sleep, such as insomnia symptoms [12] and sleep disturbances [27]. According to the results here presented, studies on job stress and self-reported insomnia symptoms should also incorporate emotional demands, particularly among nurses.

Considering the scale dimensions, the odds for self-reported insomnia symptoms were slightly higher for persons with high psychological demands than for those with high emotional demands. Additionally, positions with a lack of control and social support revealed significant higher odds for self-reported insomnia symptoms as well. These results are convincing because they are similar to the outcomes of previous research suggesting higher risk for insomnia due to high demands, low control, and social support separately [14, 29].

The present research integrated the social support dimension into the job stress models. This approach allowed confirming the assumption that social support makes a difference, which seems to be higher for the emotional demand-control model than for the job strain model as judged by differences in odds between groups with high and low social support. The results showed that the three dimensions combined (high demands, low control, and low social support) increased the chances of self-reported insomnia symptoms. Furthermore, these results showed that the combination increases the odds of self-reported insomnia symptoms more significantly than each of the three dimensions separately.

Some limitations of this study should be mentioned. The study design was a cross-sectional one, which always has the disadvantage of measuring correlation rather than causality. Therefore, there is the possibility of reverse causality between job strain, lack of support, and self-reported insomnia symptoms. There is a real possibility that stress and low social support do not lead to insomnia, but that preexisting self-reported insomnia symptoms can contribute to a bad mood, which can lead to difficulties in facing demands, but also to treating colleagues poorly, which may cause the lack of social support. Moreover, some potential confounders were not included in the adjustment of the results, such as information on depression, which may influence sleep disturbance [4]. Another potential confounder is the use of sleeping pills, which has already been associated with work-related stress among female nurses [39]. Furthermore, no data on social support from family and friends, which can have a significant impact on sleep quality [40], were collected.

Among the strengths of the current research is the use of a large sample, which enabled reliable results. The inclusion of emotional demands as a psychosocial factor linked to the demand-control model allowed the exploration of this relatively new concept, thus providing an original contribution to the knowledge of work-related sleep disturbances.

Besides approaching the “classic” job strain model of psychological demands and job control, this study took emotional demands and social support into account. Both aspects are vitally important for occupations like nursing, which require caring personal service [41]. This is one of the professions where high emotional demands are caused by dealing with patients, seeing their suffering and sometimes their death, but also where one must rely on one's team, coworkers, and supervisors in order to do the job professionally. Therefore, it is important to include both aspects in the association between job stress and self-reported insomnia symptoms. Although the precise directional relationship between job stress, social support, and self-reported insomnia symptoms in this study is unclear, it is advisable to perform certain stress management and team building activities to improve individual coping stress strategies, the collective work climate, and solidarity in the workforce. These measures would benefit workers' health, the hospitals themselves through the quality of assistance, and the economy in general.

## Figures and Tables

**Table 1:** Description of the whole group; bivariate analysis between sample characteristics and self-reported insomnia symptoms, based on chi-square tests. Rio de Janeiro, Brazil, 2011.

Sample characteristics	Whole group		Without insomnia symptoms		With insomnia symptoms		*p*
	*n*	%	*n*	%	*n*	%	
Age							0.003
≤34 years	1153	35.7	799	38.1	354	30.9	
35–54 years	1755	54.4	1131	53.9	624	35.9	
≥55 years	277	8.6	167	8.0	110	40.1	
Sex							0.013
Male	411	12.7	294	13.8	117	28.8	
Female	2818	87.3	1839	86.2	979	35.1	
Race							0.730
Black	347	10.7	222	10.6	125	36.2	
White	1775	55.0	1181	56.2	594	33.7	
Mixed	956	29.6	624	29.7	332	35.2	
Others	113	3.5	73	3.5	40	35.7	
Per capita income per month							0.070
≤US$1,740	877	27.2	559	27.8	318	36.5	
US$1,741–US$3,050	1194	37.0	783	38.9	411	34.9	
≥US$3,051	978	30.3	671	33.3	307	31.6	
Education level							0.224
University degree	795	24.6	537	25.6	258	33.0	
Postgraduation	2165	67.0	1403	66.8	762	35.4	
Master's or PhD	231	7.2	160	7.6	71	30.9	
Marital status							0.016
Married/cohabiting	1833	56.8	1180	56.1	653	36.1	
Divorced/separated	593	18.4	387	18.4	206	35.5	
Never married/cohabiting	764	23.7	535	25.5	229	30.1	
Children < 6 years							<0.001
Yes	587	18.2	349	16.7	238	40.7	
No	2583	80.0	1740	83.3	843	33.0	
Working hours							0.032
<40 hours	753	23.3	472	21.5	281	37.5	
40–59 hours	1565	48.5	1277	58.1	288	31.6	
≥60 hours	789	24.4	449	20.4	340	33.0	
Night work							0.085
Current night work	1979	61.3	1329	62.3	650	33.2	
Former night work	1036	32.1	657	30.8	379	37.0	
Never night work	214	6.6	147	6.9	67	31.8	
BMI							0.004
Underweight	39	1.2	21	1.0	18	46.2	
Normal weight	1426	44.2	982	47.9	444	31.2	
Overweight	1001	31.0	654	31.9	347	35.0	
Obese	633	19.6	391	19.1	242	38.4	
Physical activity							<0.001
Yes	1017	31.5	726	34.3	291	28.8	
No	2182	67.6	1389	65.7	793	36.7	
Risk of alcohol consumption							0.009
No risk	1083	33.5	679	40.1	404	34.1	
Low risk	1563	48.4	961	56.8	602	33.3	
High risk	119	3.7	53	3.1	66	45.8	
Smoking habits							<0.001
Nonsmoker	2424	75.1	1637	77.4	787	32.8	
Ex-smoker	501	15.5	319	15.1	182	36.5	
Smoker	281	8.7	160	7.6	121	43.5	
Coffee intake							<0.001
No coffee	672	20.8	464	22.1	208	31.1	
≤3 cups per day	1452	45.0	988	47.0	464	32.2	
>3 cups per day	1057	32.7	648	30.9	409	39.2	

**Table 2:** Crude and adjusted OR for the association between job strain dimensions and self-reported insomnia symptoms, based on multivariate logistic regression tests. Rio de Janeiro, Brazil, 2011.

Job stress model dimensions	With insomnia symptoms		OR crude	Multivariate model 1	Multivariate model 2	Multivariate model 3
	*n*	%				
Psychological demands						
Low	274	29.4	1.0	1.0	1.0	1.0
Medium	339	32.8	1.24 (1.01–1.51)	1.33 (1.08–1.65)	1.33 (1.08–1.65)	1.33 (1.07–1.64)
High	451	40.0	1.68 (1.38–2.05)	1.90 (1.54–2.34)	1.89 (1.54–2.34)	1.82 (1.48–2.27)
Emotional demands						
Low	242	28.3	1.0	1.0	1.0	1.0
Medium	326	33.3	1.24 (0.99–1.53)	1.20 (0.97–2.07)	1.20 (0.97–1.50)	1.19 (0.96–1.49)
High	512	39.5	1.60 (1.31–1.95)	1.69 (1.38–2.07)	1.69 (1.38–2.07)	1.59 (1.29–1.96)
Job control						
High	334	32.4	1.0	1.0	1.0	1.0
Medium	345	32.3	1.00 (0.82–1.21)	0.98 (0.80–1.19)	0.99 (0.80–1.20)	1.00 (0.82–1.22)
Low	372	39.8	1.36 (1.11–1.65)	1.36 (1.11–1.66)	1.37 (1.12–1.68)	1.40 (1.14–1.72)
Social support						
High	311	29.4	1.0	1.0	1.0	1.0
Medium	397	34.1	1.17 (0.96–1.41)	1.14 (0.94–1.38)	1.14 (0.94–1.39)	1.18 (0.97–1.44)
Low	365	41.1	1.58 (1.29–1.92)	1.58 (1.29–1.93)	1.58 (1.29–1.93)	1.59 (1.29–1.95)

Multivariate model 1: adjustments for sociodemographics: sex, age, income, marital status, and children under 6 years.

Multivariate model 2: model 1 + adjustments for work-related factors: working hours and night work.

Multivariate model 3: model 2 + adjustments for health-related factors: BMI, smoking habits, alcohol consumption, physical activity, and coffee intake.

**Table 3:** Crude and adjusted OR for the association between Demand-Control-Support Models, and self-reported insomnia symptoms, based on multivariate logistic regression tests. Rio de Janeiro, Brazil, 2011.

Models of job stress	With insomnia symptoms		OR crude	Multivariate model 1	Multivariate model 2	Multivariate model 3
	*n*	%				
Job strain						
Low-strain	231	27.4	1.0	1.0	1.0	1.0
Passive	206	32.3	1.32 (1.04–1.68)	1.32 (1.03–1.68)	1.33 (1.04–1.70)	1.33 (1.04–1.71)
Active	269	36.5	1.63 (1.30–2.05)	1.75 (1.38–2.20)	1.75 (1.39–2.21)	1.69 (1.33–2.15)
High-strain	327	42.4	1.99 (1.60–2.49)	2.20 (1.75–2.77)	2.22 (1.76–2.80)	2.20 (1.74–2.78)
Job strain + social support						
Low-strain + high support	386	29.7	1.0	1.0	1.0	1.0
Low-strain + low support	314	35.0	1.19 (0.82–1.17)	1.17 (0.81–1.70)	1.19 (0.82–1.72)	1.17 (0.80–1.71)
High-strain + high support	90	36.0	1.62 (1.16–2.26)	1.81 (1.28–2.55)	1.83 (1.29–2.58)	1.78 (1.26–2.52)
High-strain + low support	235	45.6	2.40 (1.84–3.14)	2.60 (1.98–3.42)	2.64 (2.00–3.48)	2.64 (1.99–3.49)
Emotional demand-control model						
Low demand + high control	276	29.6	1.0	1.0	1.0	1.0
Low demand + low control	274	33.3	1.18 (0.95–1.47)	1.18 (0.94–1.47)	1.19 (0.95–1.48)	1.20 (0.96–1.51)
High demand + high control	231	34.7	1.25 (0.99–1.56)	1.32 (1.05–1.66)	1.32 (1.04–1.66)	1.25 (0.99–1.58)
High demand + low control	266	44.9	1.89 (1.50–2.37)	2.08 (1.64–2.62)	2.08 (1.65–2.63)	1.99 (1.57–2.53)
Emotional demand-control model + social support						
Low demand/high control + high support	416	30.3	1.0	1.0	1.0	1.0
Low demand/low control + low support	358	35.1	1.10 (0.79–1.5)	1.10 (0.80–1.54)	1.12 (0.81–1.56)	1.13 (0.81–1.59)
High demand/high control + high support	69	36.3	1.32 (0.92–1.90)	1.48 (1.02–2.15)	1.49 (1.02–2.16)	1.41 (0.96–2.05)
High demand/low control + low support	197	49.4	2.33 (1.77–3.07)	2.53 (1.91–3.35)	2.55 (1.93–3.39)	2.47 (1.86–3.29)

Multivariate model 1: adjustments for sociodemographics: sex, age, income, marital status, and children under 6 years.

Multivariate model 2: model 1 + adjustments for work-related factors: working hours and night work.

Multivariate model 3: model 2 + adjustments for health-related factors: BMI, smoking habits, alcohol consumption, physical activity, and coffee intake.

## References

[1] Cunnington D., Junge M. F., Fernando A. T. (2013). Insomnia: prevalence, consequences and effective treatment. *The Medical journal of Australia*.

[2] Doghramji P. P. (2014). Integrating modern concepts of insomnia and its contemporary treatment into primary care. *Postgraduate Medicine*.

[3] Irish L. A., Dougall A. L., Delahanty D. L., Hall M. H. (2013). The impact of sleep complaints on physical health and immune outcomes in rescue workers: a 1-year prospective study. *Psychosomatic Medicine*.

[4] Fernandez-Mendoza J., Shea S., Vgontzas A. N., Calhoun S. L., Liao D., Bixler E. O. (2015). Insomnia and incident depression: role of objective sleep duration and natural history. *Journal of Sleep Research*.

[5] Li Y., Vgontzas A. N., Fernandez-Mendoza J. (2015). Insomnia with physiological hyperarousal is associated with hypertension. *Hypertension*.

[6] Sands-Lincoln M., Loucks E. B., Lu B. (2013). Sleep duration, insomnia, and coronary heart disease among postmenopausal women in the women's health initiative. *Journal of Women's Health*.

[7] Gadinger M. C., Fischer J. E., Schneider S., Fischer G. C., Frank G., Kromm W. (2009). Female executives are particularly prone to the sleep-disturbing effect of isolated high-strain jobs: a cross-sectional study in German-speaking executives. *Journal of Sleep Research*.

[8] Lopes C. S., Robaina J. R., Rotenberg L., Sahoo S. (2012). Epidemiology of insomnia: prevalence and risk factors. *Can't Sleep? Issues of Being an Insomniac*.

[9] Palm A., Janson C., Lindberg E. (2015). The impact of obesity and weight gain on development of sleep problems in a population-based sample. *Sleep Medicine*.

[10] Thakkar M. M., Sharma R., Sahota P. (2014). Alcohol disrupts sleep homeostasis. *Alcohol*.

[11] Kurotani K., Kochi T., Nanri A. (2015). Dietary patterns and sleep symptoms in Japanese workers: the Furukawa Nutrition and Health Study. *Sleep Medicine*.

[12] Yoshioka E., Saijo Y., Kita T., Satoh H., Kawaharada M., Kishi R. (2013). Effect of the interaction between employment level and psychosocial work environment on insomnia in male Japanese public service workers. *International Journal of Behavioral Medicine*.

[13] Åkerstedt T., Fredlund P., Gillberg M., Jansson B. (2002). Work load and work hours in relation to disturbed sleep and fatigue in a large representative sample. *Journal of Psychosomatic Research*.

[14] Kim H.-C., Kim B.-K., Min K.-B., Min J.-Y., Hwang S.-H., Park S.-G. (2011). Association between job stress and insomnia in Korean workers. *Journal of Occupational Health*.

[15] Nomura K., Nakao M., Takeuchi T., Yano E. (2009). Associations of insomnia with job strain, control, and support among male Japanese workers. *Sleep Medicine*.

[16] de Lange A. H., Kompier M. A. J., Taris T. W. (2009). A hard day's night: a longitudinal study on the relationships among job demands and job control, sleep quality and fatigue. *Journal of Sleep Research*.

[17] Ota A., Masue T., Yasuda N. (2009). Psychosocial job characteristics and insomnia: a prospective cohort study using the Demand-Control-Support (DCS) and Effort-Reward Imbalance (ERI) job stress models. *Sleep Medicine*.

[18] Karasek R., Brisson C., Kawakami N., Houtman I., Bongers P., Amick B. (1998). The Job Content Questionnaire (JCQ): an instrument for internationally comparative assessments of psychosocial job characteristics. *Journal of Occupational Health Psychology*.

[19] Karasek R., Theorell T. (1990). *Healthy Work—Stress, Productivity, and the Reconstruction of Working Life*.

[20] De Jonge J., Van Vegchel N., Shimazu A., Schaufeli W., Dormann C. (2010). A longitudinal test of the demand-control model using specific job demands and specific job control. *International Journal of Behavioral Medicine*.

[21] Kristensen T. S., Hannerz H., Høgh A., Borg V. (2005). The Copenhagen Psychosocial Questionnaire—a tool for the assessment and improvement of the psychosocial work environment. *Scandinavian Journal of Work, Environment and Health*.

[22] de Jonge J., Le Blanc P. M., Peeters M. C. W., Noordam H. (2008). Emotional job demands and the role of matching job resources: a cross-sectional survey study among health care workers. *International Journal of Nursing Studies*.

[23] Johannessen H. A., Gravseth H. M., Sterud T. (2015). Psychosocial factors at work and occupational injuries: a prospective study of the general working population in Norway. *American Journal of Industrial Medicine*.

[24] van Vegchel N., de Jonge J., Söderfeldt M., Dormann C., Schaufeli W. (2004). Quantitative versus emotional demands among Swedish human service employees: moderating effects of job control and social support. *International Journal of Stress Management*.

[25] Gonge H., Jensen L. D., Bonde J. P. (2001). Do psychosocial strain and physical exertion predict onset of low-back pain among nursing aides?. *Scandinavian Journal of Work, Environment and Health*.

[26] Zapf D., Seifert C., Schmutte B., Mertini H., Holz M. (2001). Emotion work and job stressors and their effects on burnout. *Psychology and Health*.

[27] Theorell T., Perski A., Akerstedt T. (1988). Changes in job strain in relation to changes in physiological state: a longitudinal study. *Scandinavian Journal of Work, Environment and Health*.

[28] Ota A., Masue T., Yasuda N., Tsutsumi A., Mino Y., Ohara H. (2005). Association between psychosocial job characteristics and insomnia: an investigation using two relevant job stress models—the demand-control-support (DCS) model and the effort-reward imbalance (ERI) model. *Sleep Medicine*.

[29] Nishitani N., Sakakibara H. (2010). Job stress factors, stress response, and social support in association with insomnia of japanese male workers. *Industrial Health*.

[30] Pelfrene E., Vlerick P., Kittel F., Mak R. P., Kornitzer M., De Backer G. (2002). Psychosocial work environment and psychological well-being: assessment of the buffering effects in the job demand-control(-support) model in BELSTRESS. *Stress and Health*.

[31] Jarrin D. C., Chen I. Y., Ivers H., Morin C. M. (2014). The role of vulnerability in stress-related insomnia, social support and coping styles on incidence and persistence of insomnia. *Journal of Sleep Research*.

[32] Winwood P. C., Lushington K. (2006). Disentangling the effects of psychological and physical work demands on sleep, recovery and maladaptive chronic stress outcomes within a large sample of *Australian nurses*. *Journal of Advanced Nursing*.

[33] Burgard S. A., Ailshire J. A. (2009). Putting work to bed: stressful experiences on the job and sleep quality. *Journal of Health and Social Behavior*.

[34] Loft M., Cameron L. (2014). The importance of sleep: relationships between sleep quality and work demands, the prioritization of sleep and pre-sleep arousal in day-time employees. *Work and Stress*.

[35] de Araújo T. M., Karasek R. (2008). Validity and reliability of the job content questionnaire in formal and informal jobs in Brazil. *Scandinavian Journal of Work, Environment and Health, Supplement*.

[36] National Institute on Alcohol Abuse and Alcoholism (NIAAA) http://www.niaaa.nih.gov/alcohol-health/overview-alcohol-consumption/moderate-binge-drinking.

[37] Felitti V. J., Anda R. F., Nordenberg D. (1998). Relationship of childhood abuse and household dysfunction to many of the leading causes of death in adults: the adverse childhood experiences (ACE) study. *American Journal of Preventive Medicine*.

[38] Magnago T. S. B., Lisboa M. T. L., Griep R. H., Zeitoune R. C., Tavares J. P. (2010). Condições de trabalho de profissionais da enfermagem: avaliação baseada no modelo demanda-controle. *Acta Paulista de Enfermagem*.

[39] Dorrian J., Paterson J., Dawson D., Pincombe J., Grech C., Rogers A. E. (2011). Sleep, stress and compensatory behaviors in Australian nurses and midwives. *Revista de Saúde Pública*.

[40] Ailshire J. A., Burgard S. A. (2012). Family relationships and troubled sleep among U.S. adults: examining the influences of contact frequency and relationship quality. *Journal of Health and Social Behavior*.

[41] Stansfeld S. A., Pike C., McManus S. (2013). Occupations, work characteristics and common mental disorder. *Psychological Medicine*.

